# Intraoperative monitoring of neuromuscular function with soft, skin-mounted wireless devices

**DOI:** 10.1038/s41746-018-0023-7

**Published:** 2018-05-23

**Authors:** Yuhao Liu, Limei Tian, Milan S. Raj, Matthew Cotton, Yinji Ma, Siyi Ma, Bryan McGrane, Arjun V. Pendharkar, Nader Dahaleh, Lloyd Olson, Haiwen Luan, Orin Block, Brandon Suleski, Yadong Zhou, Chandrasekaran Jayaraman, Tyler Koski, A. J. Aranyosi, John A. Wright, Arun Jayaraman, Yonggang Huang, Roozbeh Ghaffari, Michel Kliot, John A. Rogers

**Affiliations:** 1 Department of Materials Science and Engineering, Frederick Seitz Materials Research Laboratory, University of Illinois at Urbana-Champaign, Urbana, IL 61801 USA; 2 Beckman Institute for Advanced Science and Technology, University of Illinois at Urbana-Champaign, Urbana, IL 61801 USA; 3 MC10 Inc., Lexington, MA 02421 USA; 4 Department of Neurosurgery, Northwestern Memorial Hospital, Chicago, IL 60611 USA; 5 Department of Engineering Mechanics, AML, Center for Mechanics and Materials, Tsinghua University, 100084 Beijing, China; 6 Department of Civil and Environmental Engineering, Mechanical Engineering, and Materials Science and Engineering, Northwestern University, Evanston, IL 60208 USA; 7 Department of Neurosurgery, Stanford University School of Medicine, Stanford, CA 94305 USA; 8 Department of Engineering Mechanics, Southeast University, 210096 Nanjing, China; 9 Max Nader Lab for Rehabilitation Technologies and Outcomes Research, Center for Bionic Medicine, Rehabilitation Institute of Chicago, Chicago, IL 60611 USA; 10 Departments of Physical Medicine & Rehabilitation and Medical Social Sciences, Northwestern University, Chicago, IL USA; 11 Center for Bio-Integrated Electronics, Departments of Materials Science and Engineering, Biomedical Engineering, Chemistry, Mechanical Engineering, Electrical Engineering and Computer Science, Neurological Surgery, Simpson Querrey Institute for Nano/Biotechnology, McCormick School of Engineering, Feinberg School of Medicine, Northwestern University, Evanston, IL 60208 USA

**Keywords:** Medical research, Neurology

## Abstract

Peripheral nerves are often vulnerable to damage during surgeries, with risks of significant pain, loss of motor function, and reduced quality of life for the patient. Intraoperative methods for monitoring nerve activity are effective, but conventional systems rely on bench-top data acquisition tools with hard–wired connections to electrode leads that must be placed percutaneously inside target muscle tissue. These approaches are time and skill intensive and therefore costly to an extent that precludes their use in many important scenarios. Here we report a soft, skin-mounted monitoring system that measures, stores, and wirelessly transmits electrical signals and physical movement associated with muscle activity, continuously and in real-time during neurosurgical procedures on the peripheral, spinal, and cranial nerves. Surface electromyography and motion measurements can be performed non-invasively in this manner on nearly any muscle location, thereby offering many important advantages in usability and cost, with signal fidelity that matches that of the current clinical standard of care for decision making. These results could significantly improve accessibility of intraoperative monitoring across a broad range of neurosurgical procedures, with associated enhancements in patient outcomes.

## Wireless biosensors: easing intraoperative monitoring

A small skin-mounted biosensing device accurately and non-invasively monitors neuromuscular activity in real-time during surgery. With many surgical procedures there is a risk of nerve damage. Although this is often temporary, in some cases it can significantly affect patients’ quality of life. Existing monitoring systems that rely on the accurate placement of needle electrodes into target nerves are cumbersome and expensive. The device developed by a team led by John Rogers, at Northwestern University, and Michel Kliot, at Stanford University, can easily be accommodated to any part of the body to monitor muscle activity in response to nerve impulses and stimulation during surgery. Furthermore, it can wirelessly transmit signals of comparable quality to needle-based systems. These devices could not only increase the use of intraoperative monitoring in hospitals but also contribute to make surgery safer.

## Introduction

Injuries to peripheral nerves during surgical procedures constitute a significant source of morbidity and result in worsened quality of life for many patients.^1,2^ Iatrogenic peripheral nerve damage is a particularly common and devastating clinical entity that leads to significant pain and compromised functional outcomes.^2–4^ For example, approximately 5% of patients undergoing arthroscopic hip repair suffer from transient neuropraxia.^5,6^ Permanent damage to nerves is a well-known and debilitating health risk associated with peripheral, cranial, and spinal nerve access.^4^ In such cases, intraoperative monitoring techniques, primarily involving evoked potentials as well as stimulated and spontaneous electromyography (EMG), provide surgeons with the ability to identify and assess vulnerable nerve sites by probing nerve-muscle activities during surgery.^7–12^ Real-time monitoring strategies offer a powerful set of capabilities for surgeons that can positively affect outcomes, but existing intraoperative systems are large, expensive, and cumbersome; they include data acquisition consoles coupled to sensing electrode leads via multiple, fixed electrical connections. These platforms demand active engagement of trained technicians who are skilled in their operation and in the placement of needle electrodes, thus preventing access to neurosurgical care in settings where such tools are unavailable and/or where the associated costs cannot be supported.^13^ Furthermore, the wired interfaces create spatial complexity and limit electrode placement^14^ and the needle electrodes used for recording EMG signals cause discomfort due to penetration through the dermis,^8^ commonly leading to postoperative pain and/or soft tissue hematomas.^15,16^ The quality of the EMG signals also depends critically on precise placement of these needles. This set of clinical limitations establishes a clear need for alternative intraoperative monitoring approaches.

An ideal solution would offer multimodal measurement modalities and high signal quality, but in physical formats and functional designs that provide: (1) soft and conformal tissue interfaces compatible with skin across all regions of the body, (2) wireless communication capabilities and on-board power supplies capable of supporting operation continuously during an entire surgical procedure, (3) non-invasive electrodes that interface with the skin, without requiring penetration, specialized skin preparation procedures or precise spatial positioning, (4) easy-to-use interfaces with automated data capture, storage, and real-time analysis, and (5) path to low-cost embodiments. Here, we present a thin, soft biosensing device, referred to as “biostamp”, and demonstrate the utility of its unique features during intraoperative neurosurgical monitoring. The soft mechanical construction and advanced encapsulation strategies allow the use of the biostamp prototype across a broad range of neurosurgical procedures, which has not been demonstrated previously. Furthermore, the soft system-level mechanics enable intimate mechanical coupling of the biostamp to the skin, even on sensitive curvilinear regions of the face, where it can capture electrical activity (e.g., EMG) and movement of targeted muscle groups in response to direct electrical stimulation of nerves, in real-time during critical interventional procedures. Detailed clinical studies on multiple patients establish the performance characteristics and practical advantages of this technology relative to conventional needle and surface-EMG (s-EMG) measurement techniques during surgeries on peripheral, spinal, and cranial nerves.

## Results

### Soft, wireless, skin-integrated platform for intraoperative monitoring

**Fig. 1:**
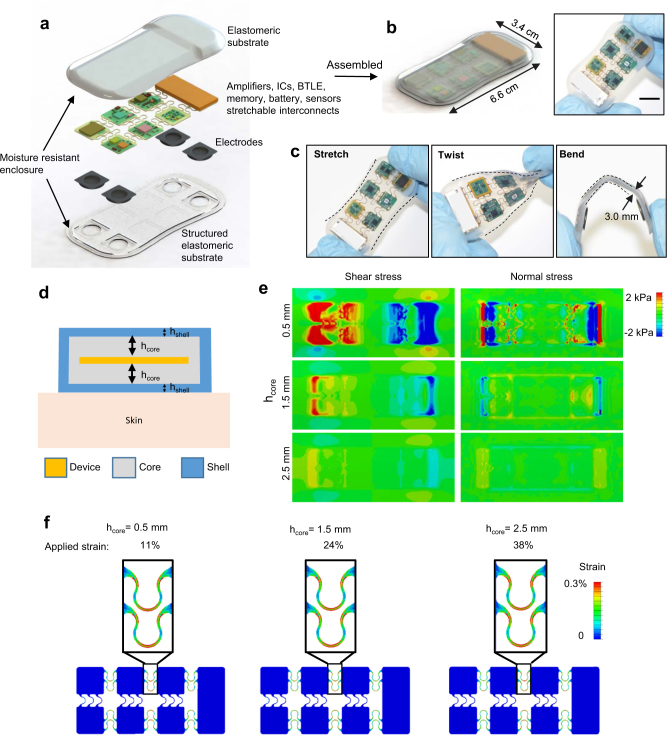
Wearable biosensing system in a soft, stretchable design. **a** Exploded view schematic illustration of the key mechanical and electrical components of the system. **b** Illustration of the biostamp fully assembled and encapsulated with soft elastomeric materials (scale bar: 1 cm). **c** Biostamp held in stretched, twisted, and bent geometries. **d** Simplified cross-sectional schematic of the electronics, core and shell encapsulation layers. **e** Computational results for interfacial stresses exerted on the skin in response to 20% tensile stretch. The shear and normal stresses vary with the thickness of the core layer (*h*_core_: 0.5, 1.5, 2.5 mm). **f** Spatial distribution of strain in the circuit components for different levels of uniaxial stretching, for different values of *h*_core_

Recent advances in materials, mechanics designs, and manufacturing methods establish the foundations for classes of thin, mechanically compliant electronic systems that enable multimodal sensing on the surface of the skin at nearly any body location.^17–19^ As demonstrated here, these platforms combine high-performance electronics and biosensors with wireless functionality to achieve high accuracy monitoring of muscle activity in response to nerve impulses and intraoperative stimulation. Figure 1a presents a schematic, exploded view of the design. The functional sub-components distribute across a collection of “islands” that interconnect electrically and mechanically via narrow, filamentary serpentine traces, optimally configured to create low modulus, “spring-like” mechanics in geometries guided by computational modeling of the mechanical and electrical characteristics. Encapsulating this “island-bridge” mesh network above and below with a low modulus, silicone elastomer defines skin-compatible physical properties, as a soft and comfortable interface to the skin (Fig. 1b). The resulting form factor and intimate skin interface are strikingly different than those of conventional wearable devices, in which rigid packaged electronic components mechanically attach to the skin via straps, penetrating pins, tapes, or bands. Stretching, twisting, bending, and other complex modes of deformation can be accommodated without altering functional operation (Fig. 1c). Robust, comfortable coupling to the skin even at tightly curved regions of the anatomy (e.g., ankle) and sensitive parts of the body (e.g., face) are possible, thereby supporting dual s-EMG and motion sensing from multiple high-flexion or contractile muscle groups, in a mode that is mechanically imperceptible to the patient and physically confined without restricting the physician.

An important enhancement of the biostamp designs used here involves embedding the active components in an ultra-low modulus silicone formulation (3–5 kPa) as a core, with a surrounding thin silicone shell that has a slightly higher modulus (50–100 kPa). This core/shell configuration allows additional degrees of freedom and motion of the serpentine interconnect structures, thereby leading to an effective modulus of 390 kPa, much smaller than that of standard designs (890 kPa) (Fig. S1). The average thickness (~2.5 mm), the physical size (3.4 × 6.6 × 0.3 cm^3^), and the weight (7 g) of such devices are similar to those of a standard gauze patch, qualitatively differentiated from any alternative commercial monitoring system currently available.

The functional electrical components include a Bluetooth^®^ Low Energy Smart radio, flash memory module (32 MB), 3-axis accelerometer and gyroscope, an analog-front end that connects to two Ag/AgCl electrodes (single lead configuration), and a rechargeable battery (15 mAhr; 10 × 25 × 1.6 mm^3^; wirelessly charging) that enables simultaneous measurement of motion and s-EMG signals for up to ~16 h. The accelerometer has an adjustable sampling rate (12–50 Hz), 2-mg sensitivity, and 12-bit resolution, sufficient to capture fine muscle movements, twitching, and spasms. The analog front-end samples s-EMG signals at 250 Hz with an amplification factor of 21.6 dB and a band-pass filter from 0.5–125 Hz. The low-end corner filtering is a byproduct of the AC-coupled relationship between the Ag/AgCl electrodes and amplifier electronics, while the high-end is set by the analog front-end to satisfy the Nyquist condition of the analog-to-digital converter (ADC).

### Quantitative analysis of the mechanical attributes

Figure 1d presents a schematic cross-sectional illustration of the design (as in Fig. 1a, b) in the core/shell configuration described above.^20^ The shell provides mechanical stability, elastic restoring force, and skin-compatible interface; the core allows the island/bridge construct to mechanically deform freely, thereby minimizing the material strains induced by system deformation. Additionally, optimized choices for the thickness of the core (Silbione, elastic modulus 3 kPa, and thickness, *h*_core_ in Fig. 1d), guided by computational mechanics, suppress stresses that can result from mechanical loading of the surface of the skin.^21^ Simulation results summarize the dependence on *h*_core_ for the case of 20% tensile strain at the system level (Fig. 1e, Fig. S1). The interfacial stresses for *h*_core_ = 0.5 mm and 1.5 mm lie below values associated with thresholds for sensation in the underlying skin (~2 kPa)^20^ in most regions; *h*_core_ = 2.5 mm enables even further reductions. Figure 1f shows the strain distributions in the first copper interconnect layer from the top, for the case of an applied strain that corresponds to the limit of elastic stretchability (with yield strain of ~0.3% in copper^22^). In all cases, the strains in the island regions are negligible. The stretchability increases with *h*_core_ (Fig. 1f) to levels comparable or larger than the elastic limit of human skin. The results in Fig. 1e and f highlight the advantages of large values of *h*_core_ (~2.5 mm). Computed results for bending appear in Figs. S2–S5. Figures S3 and S4 show the distributions of interfacial stresses on the skin for different curvatures (*κ* = 0.0092, 0.0184, and 0.0276 mm^−1^), corresponding to different bending angles (*α* = 30°, 60°, and 90°) as shown in Fig. S2. Even at large flexion angles (~90°), the interfacial stresses for *h*_core_ = 2.5 mm are below threshold values for skin sensation (~2 kPa). The corresponding strain distributions in the copper fall within the elastic limit for all three cases (i.e., *h*_core_ = 0.5, 1.5, and 2.5 mm).

### Quantitative comparisons of usability and signal fidelity to clinical standards

Current state-of-the-art intraoperative monitoring systems provide high-quality signal recordings, but they are large and complex, and the costs of the equipment and of the trained personnel necessary for its operation are prohibitive. Additionally, the wired connections and computer consoles needed to analyze and display data (Fig. S6) are cumbersome for surgeons and support staff in the operating room. A conventional system of this type (Cascade IONM) offers high sampling rates (25.6 kHz), high signal resolution (18-bit), and low noise operation (<2 µV_RMS_) optimized for EMG recordings, with penetrating needle electrodes or surface electrodes as the measurement interface. By contrast, the biostamp embeds all necessary electronics and electrodes in a single, compact platform that, itself, softly couples to the skin in a straightforward, non-invasive manner, without need for specialized skill or training, to nearly any region of the body. Although the biostamp is not hermetically sealed, the silicone encapsulation layer prevents water ingress, and thereby mitigates risks of moisture exposure to the circuitry during intraoperative monitoring procedures. The overall size (~2 orders of magnitude) and mass (~2 orders of magnitude) of the biostamp are qualitatively smaller than those of conventional hardware used for intraoperative monitoring. The wireless, battery-powered operation and intimate skin interface isolates the system from noise associated with power lines, motion artifacts, and ambient electrical interference, as demonstrated in our previous systems.^17–19^ Although sampling frequencies and levels of resolution (100 μV_RMS_, 1 kHz, and 16-bits) are somewhat lower than those of conventional systems, the quality of the data determined by signal-to-noise ratio (SNR) analysis, in practical clinical contexts, is comparable (to within ~1 dB) to that of s-EMG research tools (Delsys EMG recording system, Fig. S7). Further improvements in SNR can achieved by employing thin hydrogel layers between the skin and the recording electrodes.

### Monitoring of nerve-muscle function during surgeries on the peripheral, spine, and cranial nerves

Standard neurosurgery procedures expose targeted nerves identified for tumor removal, decompression, grafting, or other medical purposes (see “Methods” for more details). In this context, direct nerve electrical stimulation represents a common neurosurgical technique for locating, visualizing, and assessing the health of neural–muscle interfaces. Here, electrical current pulses excite nerves and polarize neuronal cell membranes, thereby producing an action potential in the nerves that leads to muscle contraction.^23,24^ The induced activation of the muscles generates a corresponding EMG response, typically recorded with penetrating needle electrodes and large-scale data acquisition systems. The minimum current that elicits a measurable EMG signal defines the stimulation current threshold. This response is strongly non-linear, with zero signal below this threshold and a smooth, monotonic increase with current above it, until saturation at some upper limiting value. With the known distance between the stimulation and measurement sites, these data allow determination of the nerve conduction velocity, a metric that highlights and characterizes nerve damage.^25^ Although threshold levels vary according to different physiological factors and the relative location of the nerves and muscle groups,^26,27^ comparison of measured thresholds serves as an effective means to compare the performance of the skin-interfaced biostamp devices reported here with clinically established tools.^28^ This approach simply quantifies the comparisons; clinical use typically involves stimulation at levels significantly above threshold.

**Fig. 2:**
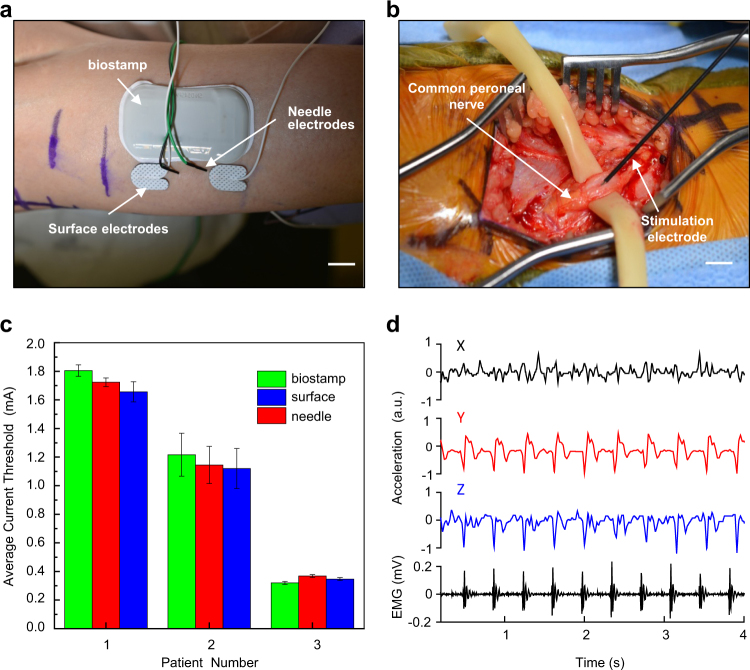
Comparative analysis of EMG recordings captured using biostamp and standard neurophysiological monitoring equipment. **a** Anatomical placement of biostamp, surface electrodes, and needle electrodes on the tibialis anterior muscle (scale bar: 1 cm). **b** Surgical access site exposing the common peroneal nerve (scale bar: 1 cm). **c** Comparison of stimulation current thresholds for the three monitoring systems determined using the configuration shown in **a**. **d** Motion and EMG waveforms recorded with biostamp during direct nerve stimulation

The clinical studies involved biostamp devices placed on one or more muscles innervated by the target nerve with consenting patients undergoing needle EMG recordings using a state-of-the-art intraoperative monitoring system during peripheral nerve, spine, or cranial nerve surgical procedures (Tables 1–3, at Northwestern Memorial Hospital). We compared measurements from the biostamp platform to those using two types of EMG electrodes with hard–wired interfaces to conventional data acquisition and conditioning electronics: needle electrodes (with Cascade IONM electronics) and surface electrodes (also with Cascade IONM electronics) (Table S1). In most perpheral surgeries, all three types of recording systemswere applied (Fig. 2a) near the surgical access point to the common peroneal nerve (Fig. 2b) for capture of EMG during stimulation.

**Table 1:** Summary of peripheral surgery patient information

Patient	Stimulated nerve	Recorded muscle	Age	Gender	Surgery
1	Right tibial nerve	Right tibialis anterior muscle	52	F	Decompression of a severe sciatic nerve stretch injury involving primarily the peroneal portion
2	Distal tibial nerve	Sole of foot muscle	19	F	Neurofibroma dissection
3	Right spinal accessory nerve	Right trapezius muscle	37	M	Neurotization with severe right brachial plexus injury
4	Right common peroneal nerve	Right peroneuslongus muscle	24	M	Decompression and neurolysis with severe right common peroneal nerve injury
5	Right tibial nerve	Right sole of foot muscle	60	M	Neurotization with laceration injury to peroneal portion of sciatic nerve in thigh
6	Right tibial nerve	Right sole of foot muscle	47	F	Schwannoma removal from right tibial nerve in the calf
7	Right C6 spinal nerve	Deltoid muscle	41	F	Schwannoma removal from the middle trunk of her right brachial plexus
8	Left common peroneal nerve	Left tibialis anterior muscle	48	M	Decompression of left common peroneal nerve with prior surgeries for treatment of a ganglion cysts
9	Right common peroneal nerve	Right tibialis anterior muscle	54	M	Decompression of right common peroneal nerve with a ganglion cyst
10	Left posterior interosseous nerve	Finger extensor digitorum muscle	45	M	Schwannoma removal from left posterior interosseous nerve

**Table 2:** Summary of spinal surgery patient information

Patient	Stimulated nerve	Recorded muscle	Age	Gender	Surgery
1	Scarred right L5 spinal nerve	Tibialis anterior muscle	30	M	Right L5/S1 spinal surgeries
2	Left L5 spinal nerve	Tibialis anterior muscle	60	F	Corrective surgery for scoliosis
3	Left L5 spinal nerve	Tibialis anterior muscle	69	F	Lumbar decompression and fusion spine surgery
4	Scarred left L5 spinal nerve	Tibialis anterior muscle	57	M	L4/5 surgeries

**Table 3:** Summary of cranial surgery patient information

Patient	Stimulated nerve	Recorded muscle	Age	Gender	Surgery
1	7th cranial nerve (facial)	Left facial muscle	43	M	Removal of left cerebello-pontine angle epidermoid mass

As outlined in detail subsequently, such measurements revealed that stimulation current thresholds determined using the biostamp were statistically indistinguishable from those determined using Cascade electronics coupled to needle electrodes and surface electrodes (Fig. 2c). In addition to high-quality electrical measurements, the biostamp supports motion sensing with an integrated accelerometer, as a complementary data stream for detecting muscle activation, where mechanical response serves as the basis of measured signal. In Fig. 2d (top three traces), motion recorded using a tri-axis accelerometer captures small mechanical vibrations caused by contractions of the tibialis anterior muscle group. The mechanical activation profiles observed in the y-planes and z-planes align with the induced EMG response of the subjacent muscle group (Fig. 2d, bottom trace). Multimodal sensing in this manner enables tracking of both electrical and mechanical signatures of muscle activity, to provide redundancy in monitoring of muscle response and to enable compensation for motion-induced artifacts. This capability could also provide insight into the depth of anesthesia.^29–31^ In control experiments, we tested for the presence of motion or other artifacts by positioning the nerve stimulator probe above threshold at a neighboring non-neural tissue site, and showed that stimulation of non-neural tissue does not generate measurable EMG signals (Fig. S8). Although motion detection represents an area of opportunity, the work reported here focuses on EMG because of its use as the current standard of care.

**Fig. 3:**
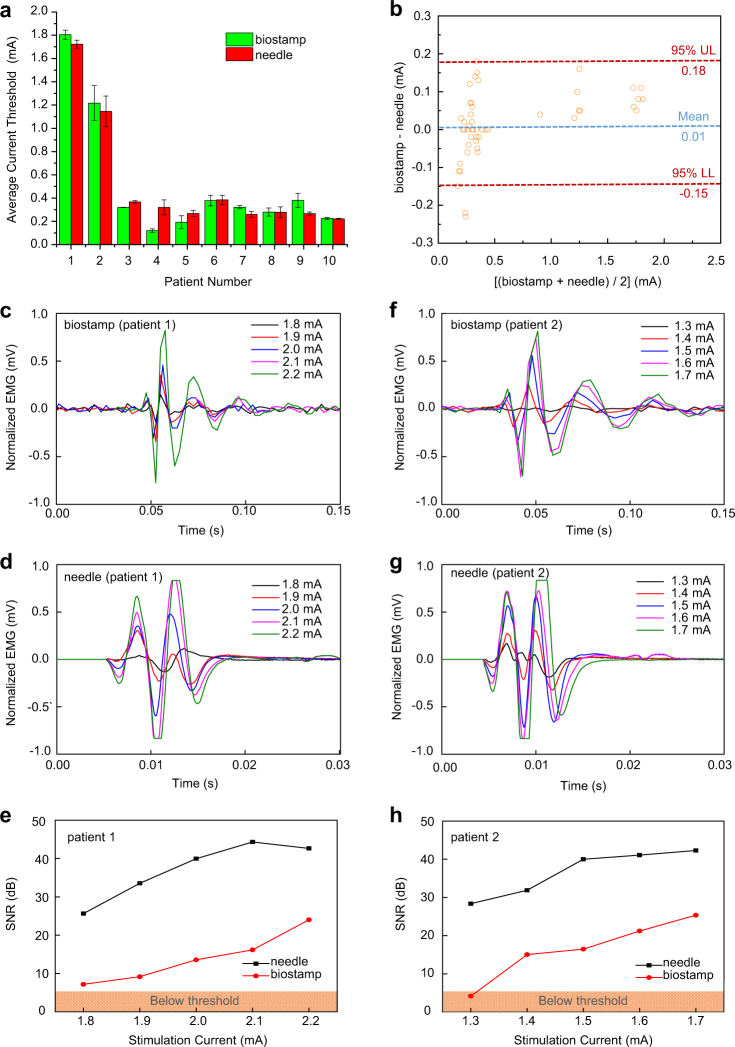
Comparison of the quality of EMG signal from the tibialis anterior muscle group captured using biostamp and conventional equipment (needle electrodes and standard recording electronics) during stimulation of the common peroneal nerve. **a** Average current thresholds determined using biostamp and conventional equipment in response to stimulation of the peroneal nerve (*n* = 10 patients). **b** Bland–Alman analysis of biostamp and conventional equipment (needle, *n* = 55 subjects) showing data sets falling within +0.18 mA (upper limit: UL) and −0.15 mA (lower limit: LL). **c**, **d** EMG signals captured using biostamp and conventional equipment, respectively, for different stimulation currents with patient 1. **e** Signal-to-noise ratio (SNR) of EMG signals shown in **c** and **d**. **f**–**h** show similar data for patient 2. For parts **c**, **d**, **f**, and **g**, EMG amplitudes (*y*-axis) correspond to normalized values

In careful comparative studies in patients undergoing peripheral nerve surgery, biostamp devices detected average current thresholds similar to those reported for conventional monitoring systems with needle electrodes (Fig. 3a; *n* = 10 patients). The precise placement of the biostamp is less susceptible to noise as the biostamp can detect average threshold currents similar to the conventional needle-based monitoring system. The biostamp and the needle-based system strongly correlated with nearly 95% of the two data sets falling within +0.18 mA and −0.15 mA (Fig. 3b), across *n* = 55 peripheral nerve surgery subjects.

This finding is important, partly because the morphology of the s-EMG waveforms captured by the biostamp had lower peak amplitudes and different decay envelopes compared to those recorded with needle electrodes in the conventional manner. The differences in amplitude varied across subjects, likely influenced by the position of the surface electrodes and the nature of the skin barrier in the case of the biostamp, and the precise placement of the needle electrodes in the case of the clinical standard. The data envelopes depend on the sampling rates and the frequency-dependent electrical impedance, both of which are different for these two systems.

Examination of representative data (patients 1 and 2) highlight some of the key findings. For patient 1, the biostamp and conventional needle platforms both exhibited current thresholds in the ~1.8–2.2 mA range, and high-quality signals with stimulation levels commonly applied during neurosurgery (Fig. 3c, d). Specifically, the EMG waveforms captured by the biostamp exhibit SNR values of ~10 dB at threshold and reach ~50 dB in response currents employed during routine neurosurgery procedures (Fig. 3e). The needle electrode system offers improved SNR, but in clinical terms, SNR thresholds above 10 dB are equivalent, in the sense that they offer routine ability for threshold detection. For patient 2, the EMG waveforms also showed robust muscle signal patterns in response to current stimulus levels in a safe operating range (Fig. 3f, g). Similar to the measurements in patient 1, the SNR levels for the needle electrode platform (~60 dB) and biostamp (~30 dB) were well above the noise at the threshold current level (biostamp: 1.4 mA, needle: 1.3 mA), and increased with current in an expected manner above threshold (1.3–1.7 mA, Fig. 3h). The differences in signal levels between these two systems largely depend on the electrode placement and stimulation location. Increasing the stimulus current resulted in increasingly large muscle responses, up to EMG amplitudes of ~0.15 mV for the biostamp and ~0.4 mV for the needle electrode platform. In both cases, the SNR was sufficiently high to detect muscle activation, throughout the normal range observed during surgical procedures.

**Fig. 4:**
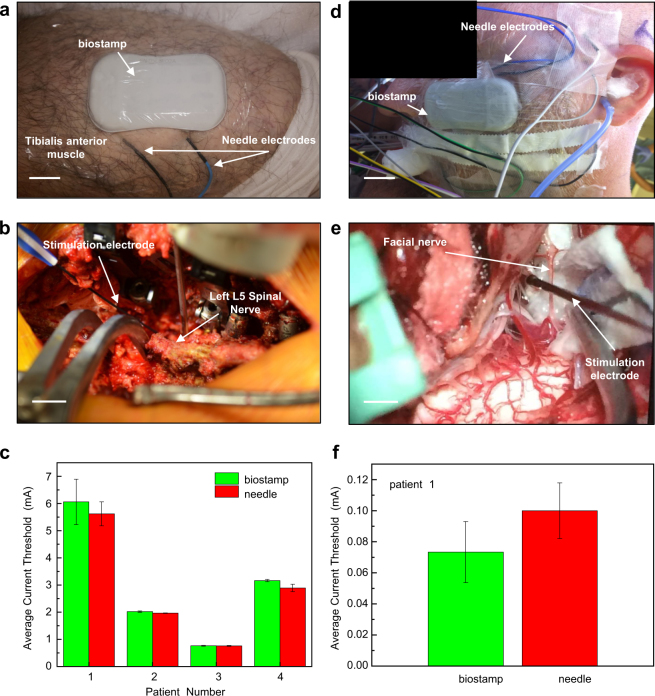
Comparison of the quality of EMG signals captured using biostamp and conventional equipment (needle electrodes and standard recording electronics) during spinal and cranial nerve surgeries. **a** Anatomical placement of biostamp and needle electrodes on the left tibialis anterior muscle (scale bar: 3 cm). **b** Surgical access site for direct stimulation of exposed left L5 spinal nerve. EMG signals were captured on the left anterior tibialis muscle (scale bar: 1 cm). **c** Average current thresholds for biostamp and conventional equipment derived from EMG signals from the tibialis anterior muscle. **d** Anatomical placement of biostamp and needle electrodes on the left facial muscle (scale bar: 2 cm). **e** Surgical access site for direct stimulation of the exposed facial nerve (scale bar: 5 cm). **f** Average current thresholds for biostamp and conventional equipment derived from EMG signals from the left facial muscle

In addition to peripheral nerve surgeries, the biostamp can provide intraoperative monitoring insights during spinal and facial nerve stimulation. As in the other cases, in spinal nerve procedures, current threshold levels for the biostamp were consistent with those of needle electrode platform (Fig. 4a–c). Across all four patients examined, threshold values determined by the two platforms are comparable, ranging from ~1–7 mA (Fig. 4c). As before, EMG waveforms measured by the biostamp and the needle electrode platform had SNRs well above the noise floor (>10 dB). In both cases, the SNR increases with stimulation current level but approached similar values above 2.3 mA (Fig. S9), indicating that the nerve fibers are activated, and thereby causing a saturated muscle response. In facial nerve procedures, the measured current thresholds using the biostamp and needle electrodes were also similar (Fig. 4d–f). Taken together, these findings demonstrate the broad applicability of the biostamp for intraoperative monitoring across multiple, highly sensitive nerve targets.

## Discussion

The results presented here demonstrate that wireless, skin-mounted device technologies that exploit soft mechanics, multifunctional electronics, and precision biosensors enable continuous electrical and mechanical monitoring of muscle responses during peripheral nerve, spine, and cranial surgeries. The ultra-soft, miniaturized form factor facilitates direct attachment to a broad range of muscle groups, including challenging regions of the anatomy such as the face. The result is a non-invasive, easy-to-use platform for capturing EMG and motion signals, with capabilities that reproduce the key functionalities of conventional, large-scale electronic platforms, which serve as the current clinical standard of care (Figs. 3, 4, Fig. S2).

Compared to the established intraoperative monitoring systems (for the purpose of this experiment we used a Cadwell Cascade IONM), the physical design of the biostamp offers significant advantages in multimodal sensing (electrophysiology and accelerometry), size, weight, and comfort, without any practically significant sacrifices in signal fidelity or threshold detectability for intraoperative monitoring. The simplicity of operation eliminates the need for personnel with specialized training in needle electrodes and EMG recording. These usability attributes, taken together, with the wireless mode of operation, offer the potential to significantly simplify clinical preparation effort and time in the operating room. In particular, the heavy demands associated with needle electrodes, the complex procedures for insertion into the tissue, and the management of wired cables that must be secured to the body of the patient and routed across the operating room table to adapter boxes and computer control systems could be greatly minimized. Figure S10 provides a series of EMG recordings obtained from the flexor carpi radialis muscle of a human subject to confirm recording stability of the biostamp over the duration of a typical surgery (<10 h). The EMG amplitude and SNRs stimulated by maximum voluntary muscle contraction in recordings remain the same (±0.2 V and 2.8 dB) during 10 h of wearing, which ensures the recording stability throughout the duration of a surgery. In addition, biostamps are placed in multiple body locations to measure electrical potentials simultaneously. Concurrent monitoring of EMG, electrocardiography, and electrooculography offers complimentary diagnostic capabilities during neurosurgical procedures (Fig. S11).

These attractive physical and operational characteristics can serve to drive procedural uptake in hospitals where complex and expensive monitoring equipment is unavailable or untenable. The ease of setup offers significant benefit to medical facilities with untrained personnel, thereby significantly broadening access to intraoperative monitoring. In addition, the soft form factor and ability for attachment to multiple body locations make this type of technology applicable across a variety of surgical procedures and clinical scenarios. For example, these platforms could be used to monitor unintended stretching of the sciatic nerve during hip surgery and the axillary nerve during shoulder surgery, both of which represent common complications of orthopedic procedures. Real-time monitoring of salient nerve health to minimize intraoperative nerve damage during parotidectomies represents another intriguing possibility. Other types of demanding surgeries where peripheral nerves are vulnerable, and where monitoring could be valuable, include procedures targeting the neck, where the spinal accessory nerve is susceptible to damage, as well as the abdomen and pelvis, where the lumbosacral plexus is at risk. The ease of use suggests the possibility for many other clinical applications of these technologies in monitoring nerve muscle integrity.

More generally, the successful application of biostamp in a surgical context foreshadows other modes of operation in different clinical use cases. Specifically, many recently reported “soft” sensor technologies^18,19,32^ can easily be incorporated into the biostamp platform to capture additional physiological parameters of interest. Examples include blood flow,^33^ blood pressure,^34,35^ temperature,^36^ hydration state,^37,38^ tissue stiffness,^39^ mechano-acoustic signatures,^17^ swelling,^40^ and many others.^41–46^ Electrical and thermal stimulation represent additional actuation possibilities. These unique sensing and actuation capabilities could enable monitoring over a broad range of surgeries beyond neurophysiological procedures. For example, in reconstructive surgical procedures, the state of health of various types of tissue flaps could be monitored by measuring blood flow in the region of the transplanted tissue and from the feeding arteries. To surveil and prevent skin pressure ulcers in immobile patients, diabetics and those with peripheral neuropathies, these added sensors and actuators could be applied to measure tissue stiffness, hydration, swelling, and temperature, providing early warning signs of tissue breakdown and ulcers. The onboard processor and wireless connectivity in combination with the biostamp sensors and actuators would ultimately allow operation in an automated mode as part of a closed loop system, which senses for skin and muscle injuries, and, in turn, delivers therapy (via drug release, thermal activation, or electrical stimulation).

In summary, soft mechanics, compact size, and wireless modes of operation in advanced skin-mounted electronic sensing technologies have the potential to fundamentally improve the state of intraoperative monitoring during neurosurgical procedures. Based on our clinical studies, the combination of biostamp measurement platforms and traditional stand-alone nerve stimulators can provide surgeons with the opportunity to monitor nerve and muscle function during a wide range of operations in which nerves are at risk of damage. Adoption of this class of wireless wearable technology may not only simplify the state of intraoperative monitoring, but also improve patient outcomes during invasive surgical procedures.

## Materials and methods

### Design of clinical study

This study was approved by the Northwestern University’s Investigational Review Board (IRB #: STU00201505) and consents were obtained from all patients prior to undergoing surgery and the scientific research study. All patients were administered general anesthesia without long-lasting paralytic agents so that muscle activity could be monitored. Once anesthetized, the biostamp devices were placed on the skin overlying relevant muscles that were also being monitored using standard needle EMG techniques. In several patients, conventional s-EMG electrodes were also used. Nerve–muscle activities were measured once target nerves were exposed surgically and direct current stimulation could be applied, as instructed and overseen by the main surgeon.

### Conventional EMG sensing electrodes and stimulation

Needle electrodes (Rhythmlink, 13-mm long, 0.4-mm diameter, 1.5-m leadwire, SP119022, stainless steel) served to monitor nerve activity during the surgeries. A stimulation probe delivered direct current to the targeted site (Prass Standard Flush-Tip Probe, Medtronic Xomed, ~10-cm long, stainless steel with plastic handle). The electrodes electrically connected to an external stimulator box (conventional intraoperative monitoring system). The stimulation pulses consisted of monophasic waveforms (at 2.6 Hz, 200 μs pulse width), at adjustable current levels with control at the level of 0.01 µA. The s-EMG recordings used hydrogel adhesive electrodes with Ag/AgCl backing layer (AMBU/Neuroline Surface Electrodes, Disposable, 700 SERIES). The distance between electrodes on the biostamp was ~5 cm. The needle and s-EMG electrodes were positioned ~5 cm apart, next to the biostamp, to facilitate comparison. The details of the data acquisition system and noise floor for the s-EMG electrodes and the needle electrodes can be found in the Table S1 and Fig. S7.

### Surgery to expose and electrically stimulate nerves while recording from muscles

The skin overlying the nerve was infiltrated superficially with local anesthetic and epinephrine. The peripheral nerve was then exposed using standard surgical techniques. Once exposed, a monopolar electrical nerve stimulation device (Prass Standard Flush-Tip Probe, Medtronic Xomed) applied electrical current at different threshold levels either directly to the surface of the nerve or to the surrounding tissues as a control (Fig. S12).

### Stimulation current threshold study

To define the thresholds, electrical stimulation was directly applied to the surface tissues of the nerve. The stimulus starts at electrical current levels that are too low to elicit activity in the studied muscle as measured by conventional EMG monitoring system. The stimulus level is then increased gradually until a muscle response could be recorded using the biostamp and the conventional intraoperative monitoring system (Fig. S7) separately. A trained intraoperative monitoring specialist at Northwestern Memorial Hospital determined the threshold current value at the point when the EMG signal waveform was visually distinguishable. This procedure was repeated six times and the stimulus threshold level for eliciting a detectable muscle response was recorded during each trial. The data were collected and then analyzed postoperatively. The reported values correspond to the average of six trials with 1 standard deviation as error bars.

### Signal recording

Needle electrode and s-EMG signals were recorded using the conventional intraoperative monitoring system under Free Run EMG mode. The built-in ADC has a sampling rate of 25.6 kHz at a gain of 50 uV/div at 18-bit resolution. The detected EMG signal was time-locked to the applied stimulation pulses. Biostamp s-EMG signals were collected with a custom engineering app interface with a sampling rate of 250 Hz and gain of 12 at 16-bit resolution.

### Signal processing

All needle and surface EMG signals were exported from the conventional intraoperative monitoring system without any data processing. Biostamp EMG signals were processed with a high pass 7th order Butterworth filter at 25 Hz. Time scales were manually aligned to synchronize the onset of nerve–muscle response from direct electrical stimulation. The SNR in dB scale is defined as 10 times log_10_(Variance^2^_Signal_/Variance^2^_Noise_) for a set period of time. All data were processed using Origin Pro software.

### Mechanical modeling and finite element analysis (FEA)

Three-dimensional FEA simulations based on commercial software packages (ABAQUS) guided optimization of the mechanics of the system. The flexible circuit model, made of polyimide (PI, elastic modulus 2.5 GPa), 11.9-μm thick Cu (elastic modulus 119 GPa), and 25.4-μm thick adhesive (elastic modulus 931 MPa), had the cross-section from top to bottom of 25.4 μm PI/Cu/12.7 μm PI/adhesive/Cu/25.4 μm PI/Cu/adhesive/12.7 μm PI/Cu/38.1 μm PI. Together with the core/shell package, the flexible circuits were mounted on a phantom skin (elastic modulus 130 kPa).

### Data deposition

All data generated or analyzed during this study are included in this published article (and its Supplementary Information files). All relevant data are available from the authors.

## Electronic supplementary material

Clinical Trial Protocol(DOC 115 kb)

Table S1(DOC 53 kb)

Supplementary Material(DOC 1094 kb)

## Electronic supplementary material

**Supplementary information** accompanies the paper on the *npj Digital Medicine* website (10.1038/s41746-018-0023-7).
