# Supplementary material for: Intraoperative monitoring of neuromuscular function with soft, skin-mounted wireless devices
Source: NPJ Digit Med. 2018 May 23;1:19. doi: 10.1038/s41746-018-0023-7 (PMC6419749; doi:10.1038/s41746-018-0023-7)
Supplement: Supplementary file 1 — Clinical Trial Protocol(DOC 115 kb) [file 41746_2018_23_MOESM1_ESM.doc]

**
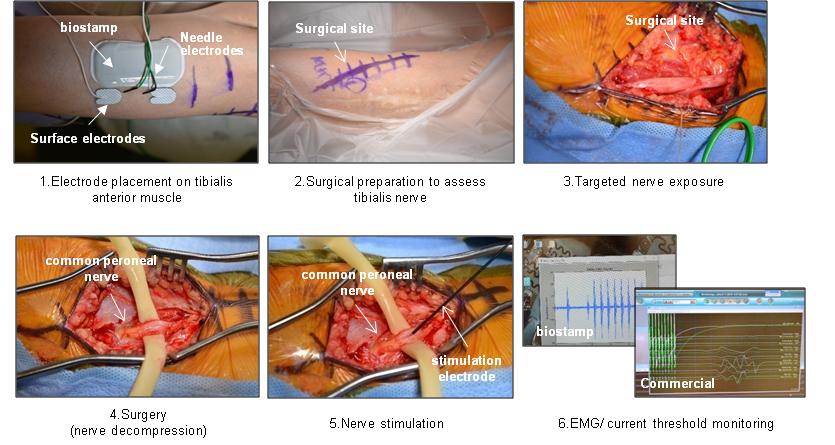
**

Nerve-muscle study procedure of patient 1 to non-invasively monitor muscle-nerve activity (using biostamp), surgically access and electrically stimulate nerves while capturing s-EMG recordings from muscles.
