# Supplementary material for: Intraoperative monitoring of neuromuscular function with soft, skin-mounted wireless devices
Source: NPJ Digit Med. 2018 May 23;1:19. doi: 10.1038/s41746-018-0023-7 (PMC6419749; doi:10.1038/s41746-018-0023-7)
Supplement: Supplementary file 2 — Table S1(DOC 53 kb) [file 41746_2018_23_MOESM2_ESM.doc]

**Table S1. Comparison of conventional intraoperative monitoring system and biostamp data acquisition systems**

| **System parameters** | **Cadwell Cascade** | **biostamp** |
| --- | --- | --- |
| *Sampling Rate* | 25.6 kHz | Up to 1 kHz |
| *Gain* | 2 - 1000 | 2 -12 |
| *Resolution* | 18 bits A/D | 16 bits A/D |
| *Bandwidth* | Up to 12.8 kHz* | Up to 500 Hz |
| *Range* | From 0.01 µV | 6 µV– 400 mV_PPK_ |
| *Analog Filter* | 2-pole high-pass,low-pass filter (12 dB/octave), and 60 Hz notch filter | - High-pass filter at 0.5 Hz   - Low-pass filter at Nyquist frequency   (e.g. 125 Hz @ f_SAMPLE_ = 250 Hz ) |
| *Noise* | < 2 µV_RMS_ | < 100 µV_RMS_ up to f_SAMPLE_ = 1 kHz |

*Bandwidth of Needle-EMG based on Nyquist rate
