# Supplementary material for: Intraoperative monitoring of neuromuscular function with soft, skin-mounted wireless devices
Source: NPJ Digit Med. 2018 May 23;1:19. doi: 10.1038/s41746-018-0023-7 (PMC6419749; doi:10.1038/s41746-018-0023-7)

Intraoperative Monitoring of Neuromuscular Function With Soft, Skin-Mounted Wireless Devices

Yuhao Liu^1^, Limei Tian^2^, Milan S. Raj^3^, Matthew Cotton^4^, Yinji Ma^5,6^, Siyi Ma^1^, Bryan McGrane^3^, Arjun V. Pendharkar^7^, Nader Dahaleh^4^, Lloyd Olson^3^, Haiwen Luan^6^, Orin Block^4^, Brandon Suleski^3^, Yadong Zhou^6,8^, Chandrasekaran Jayaraman^9,10^, Tyler Koski^4^, A. J. Aranyosi^3^, John A. Wright^3^, Arun Jayaraman^9,10^, Yonggang Huang^6,11^, Roozbeh Ghaffari^3,11^, Michel Kliot^4,7,*^, John A. Rogers^1,11,*^

^1^ Department of Materials Science and Engineering, Frederick Seitz Materials Research Laboratory, University of Illinois at Urbana-Champaign, Urbana, IL 61801, USA.

^2^ Beckman Institute for Advanced Science and Technology, University of Illinois at Urbana-Champaign, Urbana, IL 61801, USA.

^3^ MC10 Inc., Lexington, MA 02421, USA.

^4^ Department of Neurosurgery, Northwestern Memorial Hospital, Chicago, IL 60611, USA.

^5^AML, Department of Engineering Mechanics, Center for Mechanics and Materials, Tsinghua University, Beijing 100084, China.

^6^Department of Civil and Environmental Engineering, Mechanical Engineering, and Materials Science and Engineering, Northwestern University, Evanston, IL 60208, USA.

^7^Department of Neurosurgery, Stanford University School of Medicine, Stanford, CA 94305, USA.

^8^Department of Engineering Mechanics, Southeast University, Nanjing 210096, China.

^9^Max Nader Lab for Rehabilitation Technologies and Outcomes Research, Center for Bionic Medicine, Rehabilitation Institute of Chicago, Chicago, IL 60611, USA.

^10^Departments of Physical Medicine & Rehabilitation and Medical Social Sciences, Northwestern University, Chicago, IL.

^11^Center for Bio-Integrated Electronics Departments of Materials Science and Engineering, Biomedical Engineering, Chemistry, Mechanical Engineering, Electrical Engineering and Computer Science, and Neurological Surgery Simpson Querrey Institute for Nano/Biotechnology McCormick School of Engineering and Feinberg School of Medicine Northwestern University Evanston, IL 60208, USA.

*Authors share senior authorship and to whom correspondence should be addressed: [jrogers@northwestern.edu](mailto:jrogers@illinois.edu), [mkliot@stanford.edu](mailto:mkliot@stanford.edu)

Supplementary Information

**
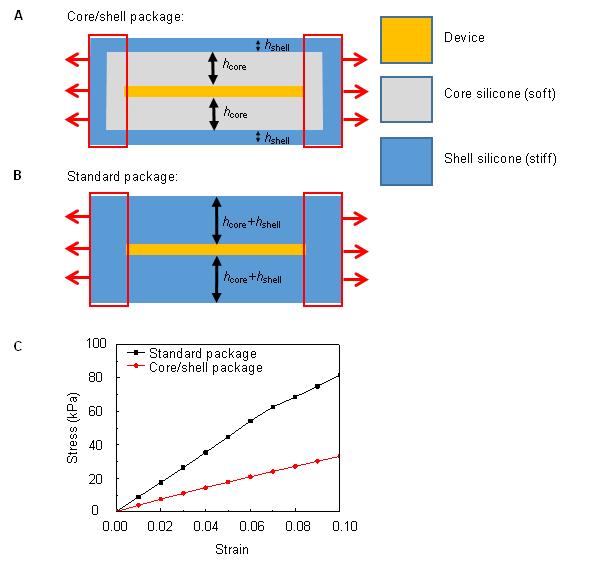
**

**Figure S1 |** Mechanics simulations for the effective modulus of biostamp with (A) core/shell and (B) standard designs. (C) Stress-strain curves measured by fixing both ends of the biostamp (not by the ends of the package) for (A) core/shell and (B) standard designs. The effective moduli were 390 kPa and 890 kPa for the core/shell and standard designs, respectively.

**
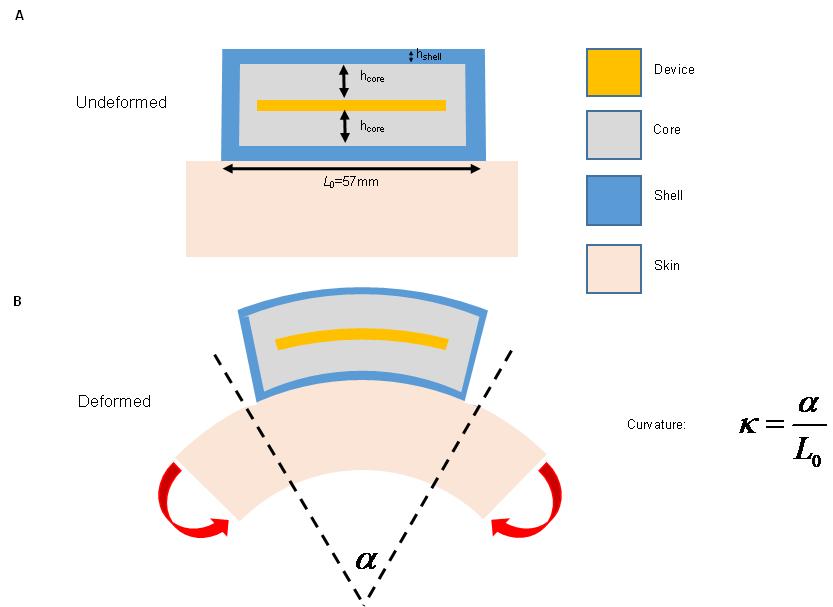
**

**Figure S2 |** Bending simulation of material layers and geometrical parameters for biostamp. (A) Schematic cross sectional illustration of biostamp in an undeformed state. (B) Biostamp undergoing bending to a defined curvature, α.

**
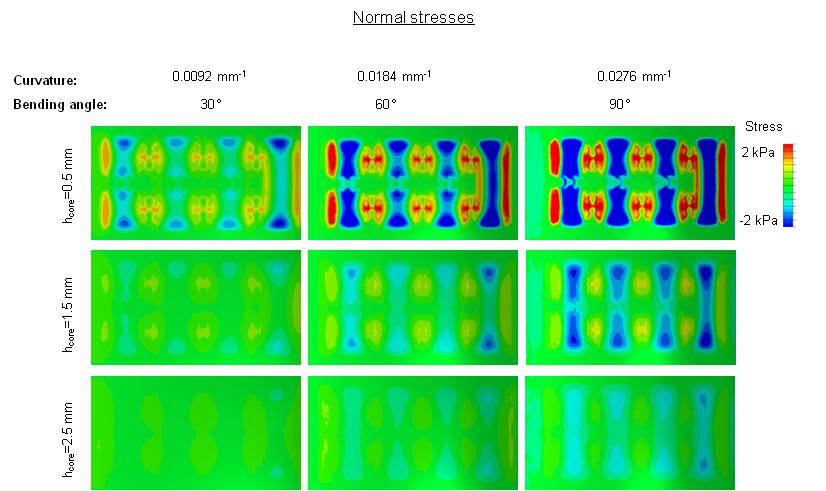
**

**Figure S3 |** Spatial distribution of normal stress induced at the interface with skin in response to varying curvatures and bending angles. The normal stresses varied with core thicknesses at 0.5 mm, 1.5 mm and 2.5 mm.


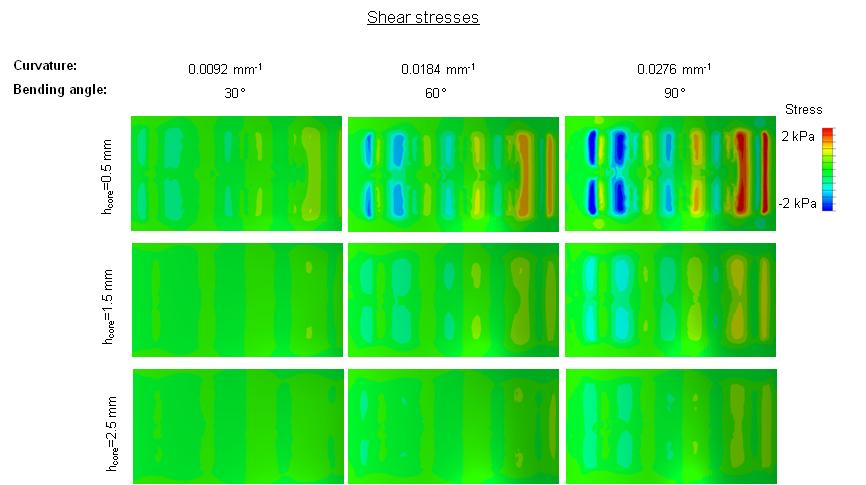


**Figure S4 |** Spatial distribution of shear stress induced at the interface with skin in response to varying curvature and bending angle. The shear stresses varied with core thicknesses at 0.5 mm, 1.5 mm and 2.5 mm.

**
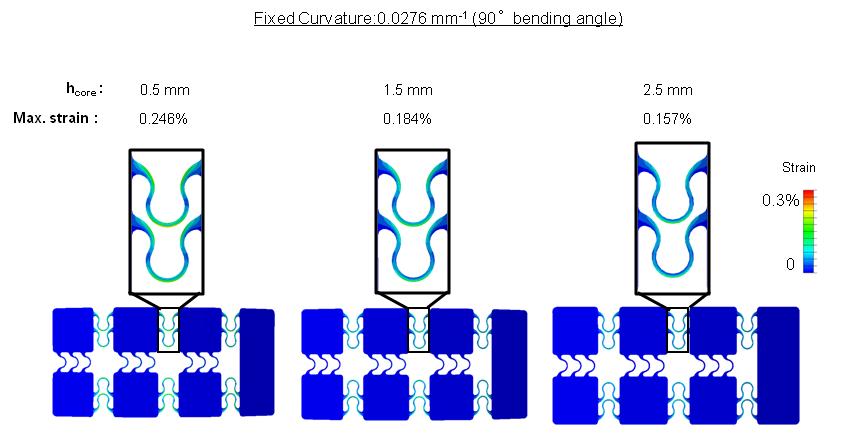
**

**Figure S5 |** Spatial distribution of strain in the circuit elements in response to a fixed curvature and bending angle for different h_core_ and the maximum strain that results.

**
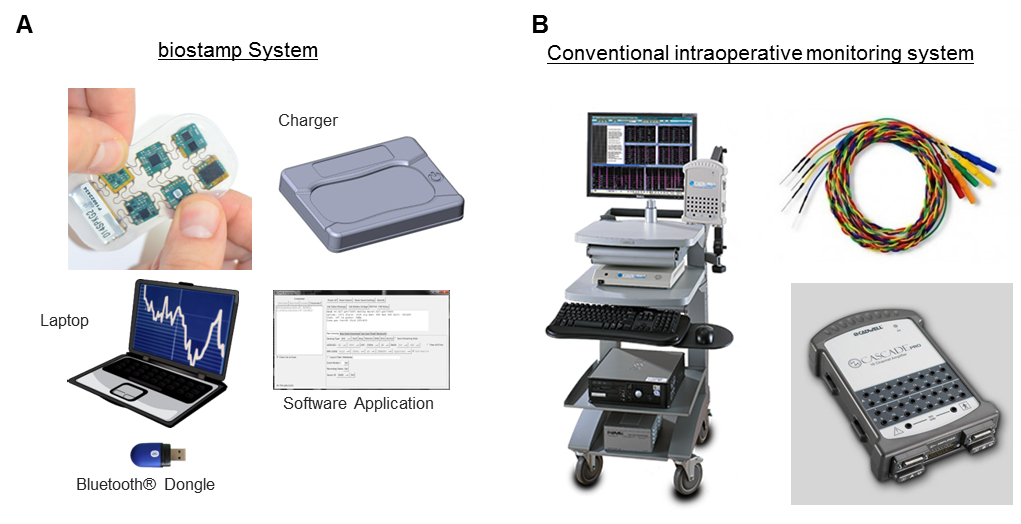
**

**Figure S6 |** Comparison of the components of the biostamp and the conventional intraoperative monitoring system. (A) Biostamp prototype device (top left), charging station (top right), laptop workstation with Bluetooth connection running a custom software application (bottom) to capture and visualize data streamed wirelessly from the device. (B) Conventional intraoperative monitoring station (left), needle electrodes with standard cable length (top right), and 16-channel adapters for electrode connections (bottom right).

**
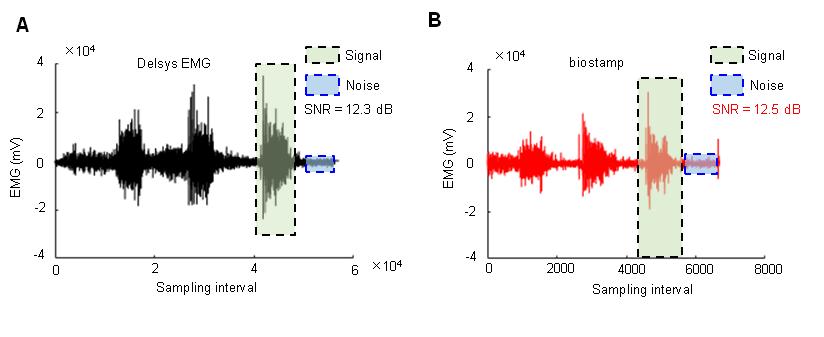
**

**Figure S7 |** Comparison of fidelity of signals captured using the biostamp and Delsys s-EMG system under voluntary muscle contraction. s-EMG waveforms from (A) biostamp and (B) Delsys system during flexion of thigh muscles. Baseline noise (at rest) is indicated for both systems. SNR estimates were comparable for biostamp (~12.5 dB) and Delsys (~12.3 dB).

**
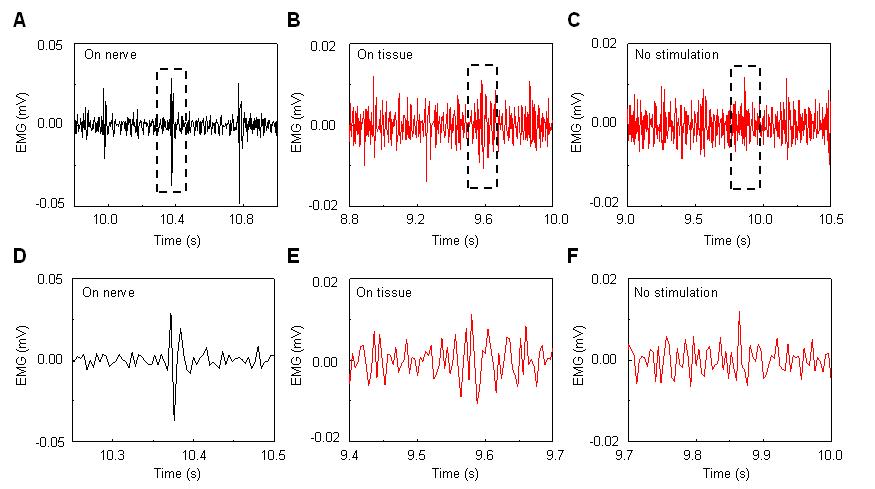
**

**Figure S8 |** Effect of stimulation artifacts on biostamp s-EMG signals. Measured s-EMG response of biostamp positioned at the tibialis anterior muscle in response to direct current simulation applied to (A) right leg tibial nerve, (B) nearby tissue, and with (C) no stimulation applied when the electrode was placed on the nerve. Magnified view of s-EMG waveforms shows distinguishable features of s-EMG in response to stimulation (D) on nerve, (E) on surrounding tissue, and (F) without stimulation.

**
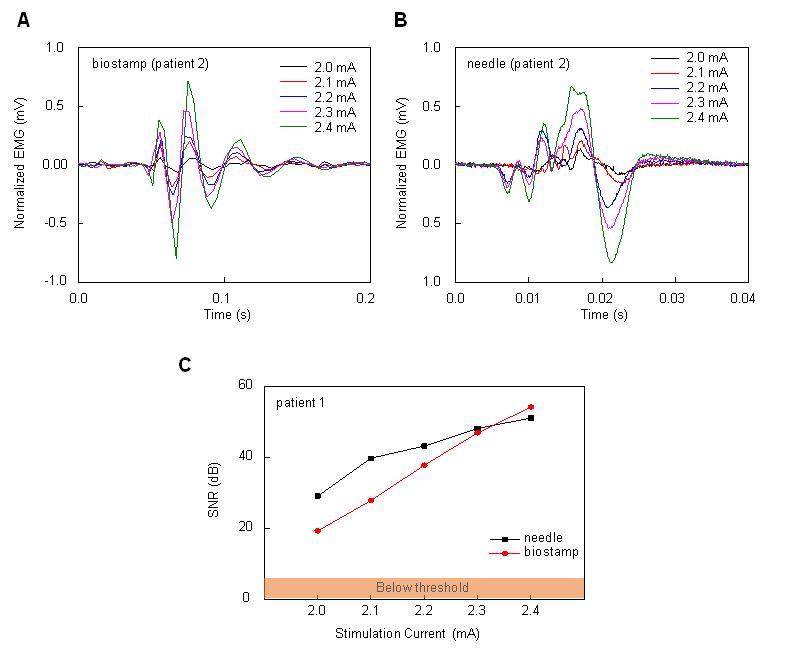
**

**Figure S9 |** Biostamps-EMG signals as function of direct nerve stimulation current during spinal surgery (patient 2 from Fig. 4C). (A) Biostamp EMG signal response as a function of direct nerve stimulation current level. (B) Commercial monitoring system with needle electrode signal response with increased direct nerve stimulation current, served as a reference to (A). (C) The signal-to-noise ratio of EMG signal response as a function of direct nerve stimulation current level in (A) and (B).


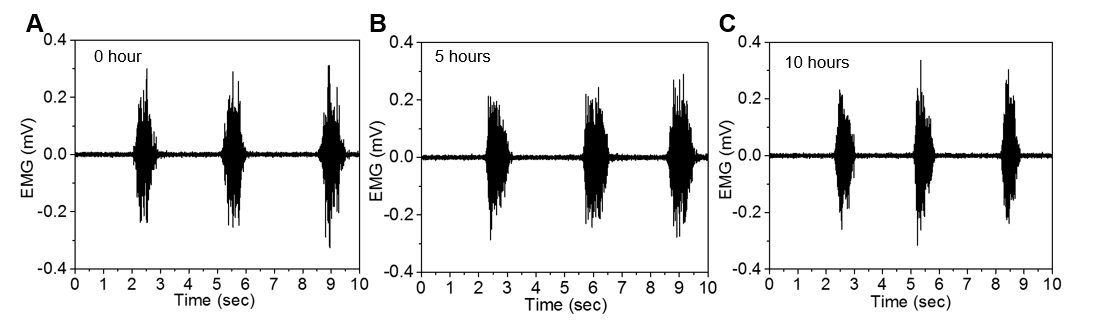


SNR: 566.3 (28 dB)

SNR: 611.6 (28 dB)

SNR: 548.5 (27 dB)

**Figure S10 |** Recording stability of biostamp. The clinical recording stability of biostamp over the duration of a typical surgery is verified by EMG recordings during 10 hours of wearing. The signal amplitude and SNRs stimulated by maximum voluntary muscle contraction remains the same.


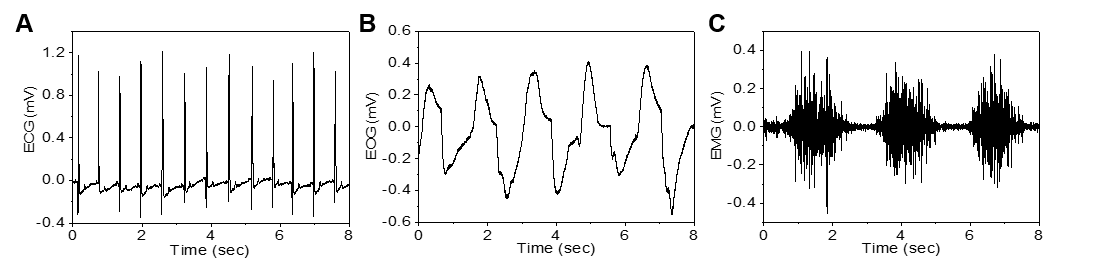


**Figure S11 |** Simultaneous operation of multiple biostamps, which measure electrocardiogram (A), electrooculogram (B) and electromyogram (C).

**
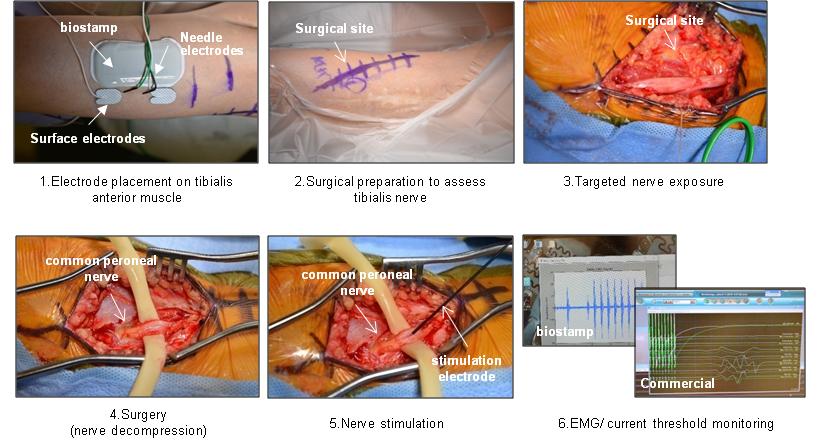
**

**Figure S12 |** Nerve-muscle study procedure of patient 1 to non-invasively monitor muscle-nerve activity (using biostamp), surgically access and electrically stimulate nerves while capturing s-EMG recordings from muscles.


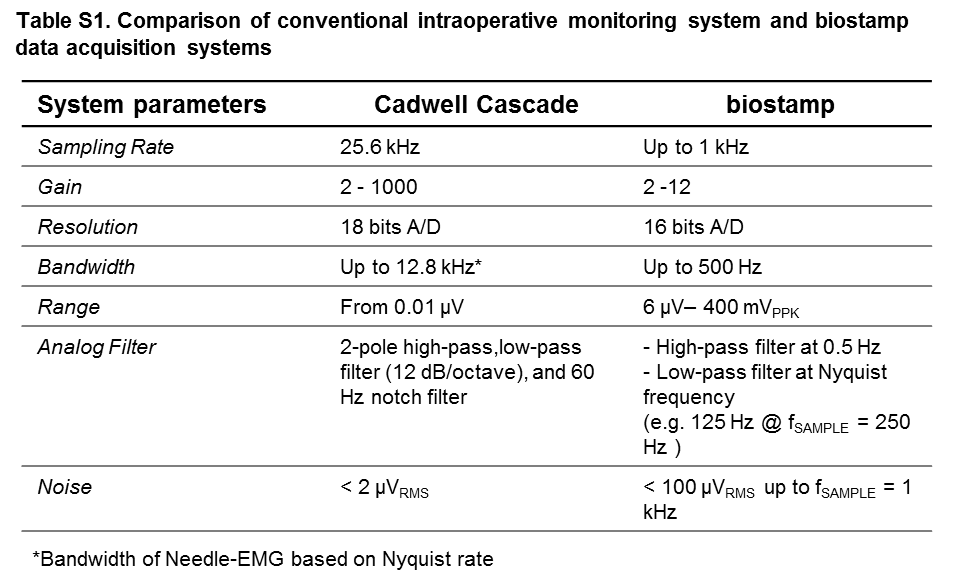

Supplement: Supplementary file 3 — Supplementary Material(DOC 1094 kb) [file 41746_2018_23_MOESM3_ESM.doc]
